# Synergistic Effects of Curcumin and 5-Fluorouracil on the Hepatocellular Carcinoma *In vivo* and *vitro* through regulating the expression of COX-2 and NF-κB

**DOI:** 10.7150/jca.41783

**Published:** 2020-04-06

**Authors:** Ting Xu, Pu Guo, Chao Pi, Yingmeng He, Hongru Yang, Yi Hou, Xianhu Feng, Qingsheng Jiang, Yumeng Wei, Ling Zhao

**Affiliations:** 1 Department of Pharmaceutics, School of Pharmacy, Southwest Medical University, No. 319, Zhongshan Rd Sanduan, Jiangyang District,Luzhou, Sichuan, 646000, P.R.China; 2 The Affiliated Hospital, Southwest Medical University, No.25, Taiping Street, Luzhou, Sichuan, 646000, China; 3 Department of Oncology, Luzhou People's Hospital, No.316, Jiugu Dadao Erduan, Luzhou, Sichuan, 646000, China; 4 School of International Education, Southwest Medical University, No.1, Xianglin Rd Yiduan, Longmatan District, Luzhou, Sichuan, 646000, China

**Keywords:** Curcumin, 5-Fluorouracil, Hepatocellular carcinoma cell lines, COX-2, NF-κB

## Abstract

Curcumin (CU) has shown broad anti-cancer effects. 5-fluorouracil (5-FU) has been a conventional chemotherapeutic agent for hepatocellular carcinoma. Unfortunately, the nonspecific cytotoxicity and multidrug resistance caused by long-term use limited the clinical efficacy of 5-FU. This study was aimed to investigate whether the combination of CU and 5-FU could generate synergistic effect in inhibiting the human hepatocellular carcinoma. The results of cytotoxicity test showed that compared with applying single drugs, the combination of CU and 5-FU (1:1, 1:2, 1:4, 2:1 and 4:1, mol/mol) presented stronger cytotoxicity in SMMC-7721, Bel-7402, HepG-2 and MHCC97H cells, while the combination groups are relatively insensitive to normal hepatocytes (L02). Among them, the molar ratio of 2:1 combination group showed strong synergistic effect in SMMC-7721cells. Then, western blotting assay further verified that the mechanism of the synergistic effect may be related to the inhibition of the expression of NF-κB (overall) and COX-2 protein. In addition, the synergistic effect was also validated in the xenograft mice *in vivo*. This research not only provides a novel and effective combination strategy for the therapy of hepatocellular carcinoma but also provides an experimental basis for the development of CU and 5-FU compound preparation.

## Introduction

Hepatocellular carcinoma (HCC) was one of the fifth major common cancers and accounted for 55% of the cases in the world 1-5. HCC was easy to metastasize and difficult to diagnose in the early stage 6, 7. Furthermore, less than 20% patients with liver cancer could be treated through surgery 8. Therefore, systemic chemotherapy became the major therapeutic means to treat liver cancer.

5-Fluorouracil (5-FU) was one of the first-line chemotherapy drugs for the treatment of malignant tumors including liver, breast and other digestive system tumors 9-11. However, the clinical application of 5-FU was limited due to its inevitable toxicity to normal cells and multidrug resistance caused by long-term use 12, 13. Recent studies have found that 5-FU combined with natural drug monomers can reduce the dose of 5-FU and increase the therapeutic effect, such as CU, sorafenib, huaier, forbesione 14-16. Among them, curcumin (CU), a natural polyphenol with low toxicity, was extracted from the rhizome of Curcuma longa Linn with a wide range of therapeutic effects especially anti-tumor property 17. Researches have elucidated that CU suppressed the proliferation of kinds of tumor cells via targeting the signal pathways including COX-2 and NF-κB 18, 19.

Recently, many experiments showed that the development of HCC could be inhibited with down-regulation of NF-κB 20, 21. Ji et al. also found that down-regulation of NF-κB could enhance the sensitivity of cancer cells to 5-FU 22. NF-κB bound to IκB kinase (IKK) then composed p50-p65-IκB tripolymer which made NF-κB in an inactive state in the cytoplasm 23. When cancer cells were activated by the activators of NF-κB, IKK dissociated and NF-κB was expressed in heterodimers and translocated from cytoplasm to nucleus, which induced the proliferation, differentiation, apoptosis and malignant metastasis of cancer cells 24. So, blocking NF-κB translocation from the cytoplasm to the nucleus was a reasonable inhibiting cancer development. COX-2 was associated with the occurrence and development of tumor through a variety of ways such as inhibiting apoptosis and stimulating the growth of tumor cells, etc 25, 26. The mechanism of anti-apoptosis associated with anti-apoptotic protein Bcl-2 which can inhibit the release of cytochrome C from mitochondria in the apoptotic HCC cells 27. Yang et al. confirmed that COX-2 was overexpression in HCC, and low expression in normal liver tissues 28. Therefore, these provided a mechanism theoretical basis for the novel combination of CU and 5-FU to treat HCC in this study.

CU and 5-FU showed synergetic effect on some cancers such as colon cancer and gastric cancer, which can not only improve the efficacy of 5-FU, but also decrease the concentration of 5-FU, preventing the damage of normal cells 29, 30. Although there were some literatures concerning about the combined effects of CU and 5-FU, their research objects or joint proportion were relatively single. In this study, we only focused on the hepatocarcinoma cell lines to study the toxicity of different combinations on different hepatoma cells and screen out the most sensitive cell line (SMMC-7721) and the best proportion (2:1). To further clarify the molecular mechanism of the combination therapy, the expression of COX-2 and NF-κB in SMMC-7721 cells were evaluated by western blotting analysis. And the synergistic effects were also validated *in vivo*. Therefore, CU combined with 5-FU is a potential method for HCC treatment in the future.

## Materials and methods

### Chemicals and reagents

CU (purity≥98%), 5-FU, RPMI 1640 medium, fetal serum, 0.25% trypsin, and 100 units/mL of penicillin-streptomycin were purchased from Thermofisher Company. MTT (3-(4,5-dimethyl-2-thiazolyl)-2,5 diphenyl-2-H-tetrazolium bromide) were purchased from Luzhou Shuangjiang Chemical Co, Ltd (Sichuan, People's Republic of China). CU and 5-FU were dissolved in DMSO (dimethyl sulfoxide) and taken as 800 μmol/L solution with complete culture solution, in which a final concentration of DMSO was 0.1% (v/v), and then further diluted as needed in cell culture medium. The NF-κBp65 antibody, COX-2 antibody and β-actin were purchased from Santa Cruz Company.

### Animals and Cell Cultures

SMMC-7721, Bel-7402, HepG-2, MHCC97H and L02 cells were obtained from Shanghai cell bank of China, and the cells were cultured in RPMI 1640 medium supplemented with 100 units/mL of penicillin-streptomycin and 10% fetal calf serum at 37°C in a 5% CO2 incubator (HEPA class100 Thermo company). Medium was replaced 3 times a week. Cells were used in the exponential growth phase for all of the experiments.

Female BALB/c nude mice aged four to six weeks (16-20 g) were obtained from Chengdu Dashuo Laboratory Animal Company (Chengdu, China), with the Laboratory Animal License: SCXK (chuan) 2015-030. The animals were fed in the IVC Animal Feeding Room of the Laboratory Animal Center of Southwest Medical University at temperature of 20 ± 2℃, with relative humidity of 40-60%. All studies on nude mice were approved by the Committee on the Ethics of Animal Experiments of the Southwest Medical University, Luzhou, People's Republic of China (No 2015DW040).

### Cell inhibition assay

To examine the inhibition effect of compound preparation of CU and 5-FU on HCC, SMMC-7721, Bel-7402, HepG-2, MHCC97H and L02 cells were inoculated into 96-well plates at the density of 5×10^3^ cells/well/100μL, respectively. After being cultured for 24 h at 37°C with 5% CO2, CU solution (6.25, 12.5, 25.00, 50.00, 100.00 and 200.00 µmol/L), 5-FU solution (6.25, 12.5, 25.0, 50.0, 100.0 and 200 µmol/L) and different combination groups of CU and 5-FU with the mole ratio of 1:1, 1:2, 2:1, 1:4, 4:1 were added to the 96-well plates with gradient concentrations and incubated for 48 h, respectively. Dimethyl sulfoxide (DMSO) was used as the solvent to prepare drug substances solutions and the same volume of solvent was considered as vehicle control. Then, 20 μL of MTT (5 mg/mL) were added to each well and the cells were incubated for another 4 h at 37 °C in the dark. The aliquots were removed and the remaining crystals (formazan precipitates) were solubilized with 150 μL of DMSO and the cells were incubated for an additional 10 min at 37 °C with gentle shaking before the measurement of the absorbance (OD) at 490 nm using an enzyme-linked immunosorbent assay. All the samples were performed in triplicate.

The tumor cell inhibitory rate was calculated as follows. Inhibitory rate (%) = (OD control - OD treated) / OD control × 100%. The IC_50_ (half maximal inhibitory concentration) values of the drugs were calculated by SPSS 18.0 software (IBM SPSS Statistics, Chicago, IL, USA).

### Examination of the Effects of Combination Agents

The median effect method was used to estimate the combination effects of the two drugs. Before the combination effect was tested, the IC_50_ (the half maximal inhibitory concentration) was determined from the exposure of the drugs including single agent and the combination agents to the HCC cells by MTT assay. And then the combination index (CI) was calculated by the following formula 31:


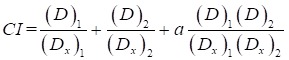


In this equation, (D)_1_ and (D)_2_ values were combined drug concentrations of CU and 5-FU, respectively, resulting in growth inhibition of the HCC (SMMC-7721 and Bel-7402) cell lines (in x%). (D_x_)_1_ and (D_x_)_2_ were the concentrations of CU and 5-FU alone that inhibited the cells growth at the same percentage (x). CI indicated synergism (CI<1), summation (CI=1) or antagonism (CI>1) of the two drugs, respectively. (D_x_)_1_ and (D_x_)_2_ can be obtained from the following formula:


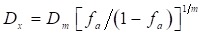


Among them, D_m_ was the median-effect dose, f_a_ was the fraction affected, and m was the slope of the median-effect plot.

### The mechanism of synergism effect of the combination of CU and 5-FU

The test was divided into eight groups including group one: curcumin alone (25 μmol/L); group two: vehicle control (RPMI1640 medium containing same concentration of DMSO); group three: 5-FU alone (15 μmol/L); group four: 2.5 μmol/L CU + 5 μmol/L 5-FU; group five: 5 μmol/L CU + 2.5 μmol/L 5-FU; group six: 5 μmol/L CU + 5 μmol/L 5-FU; group seven: 5 μmol/L CU + 10 μmol/L 5-FU; group eight: 10 μmol/L CU + 5 μmol/L 5-FU. Then, SMMC-7721 cells (up to the density of 90%) were seeded at 1×10^6^ cells into 10 cm^2^ petri dishes, then, incubated for 24 h and treated with drugs of above eight groups for 48 h, respectively. Then, the culture media were discarded and the cells were washed with cold PBS buffer twice for harvest. Cell pellets were disrupted in cell RIPA buffer and collected after centrifuging at 16000 ×g for 10 min at 4℃, and the lysates were centrifuged at 15000 ×g for 10 min at 4℃ to collect the cytoplasmic and nuclear proteins, respectively. The protein concentrations were determined by phenyl methane sulfonyl fluoride (PMSF) method. Protein samples (30 μL each) were loaded on SDS-PAGE (sodium dodecyl sulfate- polyacrylamide gels) and separated with electrophoresis and subsequently transferred onto NC membranes. Non-specific binding was blocked with 5% milk in TBST (5 mmol/L Tris-HCl, 136 mmol/L NaCl, and 0.05% Tween-20, pH 7.6) for 1 h. The membranes were cultured with primary antibodies against COX-2 (1:1000), NF-Κbp65 (1:15000) or β-actin (1:2000) overnight at 4℃. Then the membranes were washed three times with 1×TBST, followed by incubating with secondary antibodies (1:1000 dilution) at room temperature for 2 h and washed three times with 1× TBST. Protein bands were visualized by an ECL (enhanced chemiluminescence) system (Amersham Biosciences, the United States). The grey value of NF-κB, COX-2 and β-actin proteins was measured by the Quantity One software.

### *In vivo* antitumor study

#### Construction of tumor model

SMMC-7721 cells were harvested (with trypsinase) and suspended in the cell solution containing PBS buffer and Matrigel (1:1, v/v) to reach 2.5 ×10^7^ cells/mL, when the cells were in logarithmic growth phase. 0.2 mL cell suspension (5×10^6^ cells/mL) was injected subcutaneously into the right forelimb of nude mice sterilized with 75% alcohol and fed in the SPF animal room. Tumor volume (V) was measured with a vernier caliper using the formula: V = 0.5 ×a × b^2^. a was the maximum perpendicular diameter, and b was the minimum perpendicular diameter. When tumor volume reached 70-120 mm^3^, mouse models were selected for subsequent experiments.

#### Pharmacodynamics studies

Twenty tumor-bearing mice were randomly divided into four groups with 5 mice in each group. Negative control group: blank solvents including polyoxyethylene castor oil EL-35: absolute ethanol: 0.9% sodium chloride injection (1:1:6, v/v/v); CU group: CU (56.65 mg/kg); 5-FU group: 5-FU (10 mg/kg); CU/5-FU group: CU (56.65 mg/kg) + 5-FU (10 mg/kg). All groups were injected with 0.2 mL injection by intraperitoneal injection. The total treatment cycle was 29 days with the frequency of twice a week. After inoculation, tumor-bearing mice were fed and drank water freely. The diet, mental state and activities of tumor-bearing mice were observed daily. The weight of tumor-bearing mice in each group was weighed every 3 days, and the tumor volume was measured with vernier caliper.

After 48 h of the last administration, the tumor-bearing mice were sacrificed, and the tumors were removed completely. The tumor inhibition rate was calculated by weighing the tumors with an electronic balance. Tumor inhibition rate (%) = (average tumor weight of control group - average tumor weight of drug group) / average tumor weight of control group ×100% 32.

### Statistical analysis

Each experiment was performed for quintuple. Statistical data were conducted using SPSS 19.0, and summarized as means ± SD (standard deviation). The comparison between group using one-way ANOVA and Tukey test, and P-value less than 0.05 were considered statistically significant.

## Results

### Antiproliferative effects of combination group of CU and 5-FU

**Figure 1:**
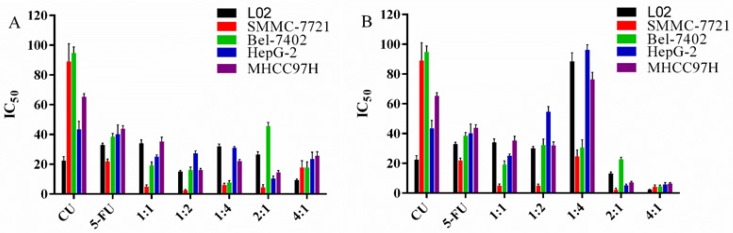
The IC_50_ value of each groups of drugs on hepatocarcinoma cells. A: The IC_50_ value of CU in each group and cell lines. B: The IC_50_ value of 5-FU in each group and cell lines.

In MTT assay, SMMC-7721, Bel-7402, HepG-2, MHCC97H and L02 cells were administrated with different concentrations of CU and 5-FU alone or in combination (1:1, 1:2, 1:4, 2:1 and 4:1, mol/mol) for 48 h, respectively. In our preliminary study, the cytotoxicities of the negative control (2‰ DMSO) did not cause significant cytotoxicity against above cell lines up to the highest dose of 200 μmol/L. All the cell viabilities were greater than 90% after incubating with 2 ‰ DMSO for 48 h (data were not shown). MTT results (Figure 1 and Table 1) showed that the growth of five kinds of cell lines were inhibited by the treatment of CU and 5-FU alone or in combinations. The cytotoxicity of all combined groups in the researched hepatoma cell lines was better than that of CU or 5-FU alone, except the combination group of 1:4 in SMMC-7721 and HepG-2 cells. Interestingly, Figure 1A-1B also showed that each combination ratio has stronger selectivity to SMMC-7721 than other three hepatoma cell lines. Furthermore, the IC_50_ of 2:1 group in SMMC-7721 cells was the least among all combination groups and cell lines. Specifically, the IC_50_ value of CU in 2:1 group was 4.32 ± 2.02 μmol/L and that of 5-FU in 2:1 group was 2.16 ± 1.05 μmol/L. Besides, compared with the toxic effect on hepatoma cells, normal liver cells line was relatively resistant to the combined treatment of CU and 5-FU with 1:1, 1:4 and 2:1 ratio. Then, the 2:1 group could selectively increase the cytotoxicity to SMMC-7721 cells. Therefore, we chose 7721 and 2:1 ratio as our subsequent research object.

### Combination effects of different proportion of CU and 5-FU in cells

**Figure 2:**
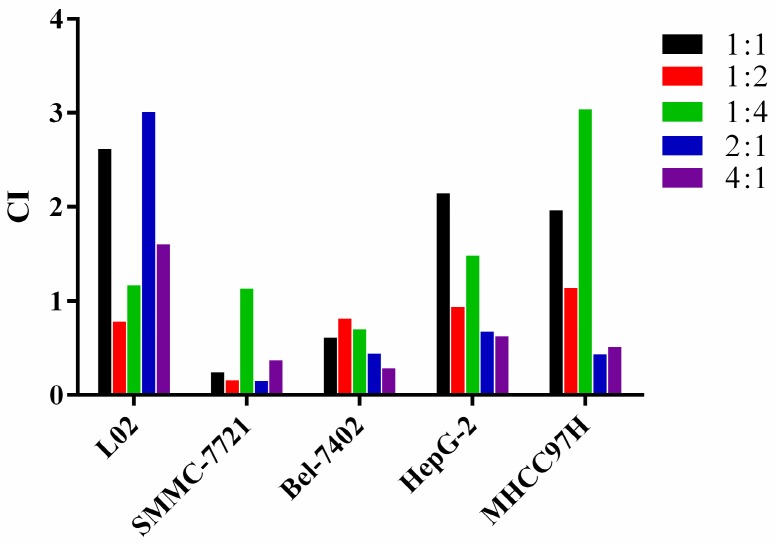
The combination effects (CI) of CU and 5-FU concurrently exposed to the HCC cell lines for 48 h. The CI values were calculated by the method mentioned above. When CI < 1, CI = 1 or CI > 1, it indicates synergism, summation or antagonism effect of the two drugs, respectively.

As shown in Figure 2 and Table 2, except that the 1:4 group (CI>1) produced antaonistic effect, most combination groups showed strong synergistic effect in 7721 cells (CI<0.3), and the 2:1 group showed the strongest synergistic effect among all cell lines. In 7402 cells, the majority combined groups showed moderate synergistic effect (0.3<CI<1). In HepG-2 cells, except for the antagonistic effect in the 1:1 and 1:4 groups, the other combined groups showed synergistic effect. In MHCC97H cells, except the 1:1, 1:2 and 1:4 groups, the other ratios showed synergistic effects. These data showed that the combination of CU and 5-FU (2:1, mol/mol) in 7721 showed the strongest synergistic inhibitory effect in all cell lines. Therefore, the 2:1 combination group and 7721 cell line were chosen as follow-up studies.

### Influence of different combination groups on SMMC-7721 cells

**Figure 3:**
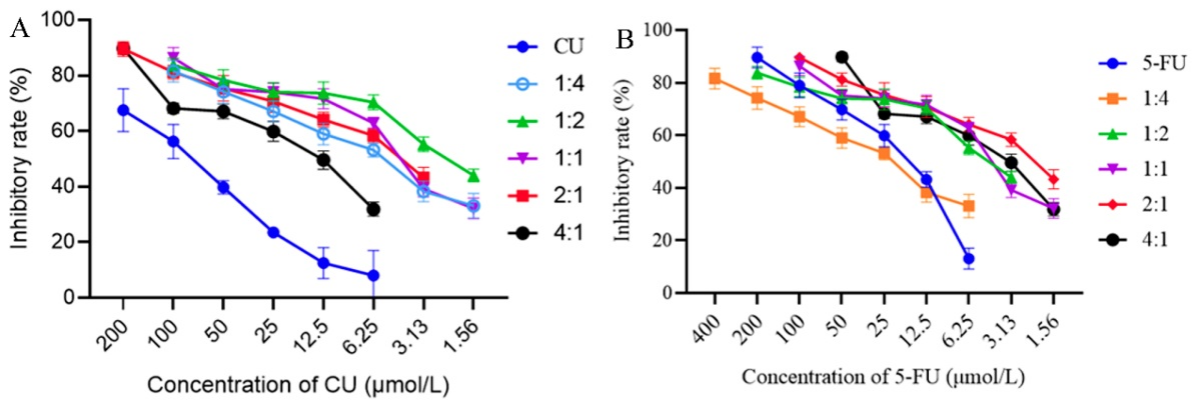
Variety of inhibition rate of combination groups in SMMC-7721 cells with the same concentration of CU (A) or 5-FU (B) after treated with 48 h. Data were expressed as means ± SD (n = 3).

In order to better understand the combined effect of CU and 5-FU in SMMC-7721 cells, a line chart of cell inhibition rate varied with proportion of CU and 5-FU was made with the constant concentration of CU or 5-FU. As shown in Figure 3B when the concentration of 5-FU was remained, at the same concentration of 5-FU, the inhibition rate of each combination was better than 5-FU single group except 1:4 combined group. Furthermore, when 5-FU was in high concentration (50-100 μmol/L), the cell inhibition effect was increased with the increase proportion of CU, especially 2:1 and 4:1 groups. When 5-FU was in medium concentration (12.5-25 μmol/L), the inhibition effect on SMMC-7721 cells decreased first and then increased with the increase proportion of CU. Among them, 2:1 group was the most effective. When 5-FU was at low concentration (6.25 μmol/L), the inhibitory effect of all combined groups was better than that of 5-FU single group. Interestingly, the combination of 2:1 showed a good inhibitory effect on SMMC-7721 cells whether in low, medium or high concentration stages. The results showed that, to a certain extent, combined with CU, the anti-cancer effect of 5-FU could be promoted.

As shown in Figure 3A, the inhibitory effect of all combination groups was stronger than CU single group, which suggested that 5-FU combined with CU could increase the anti-cancer effect of CU. Concretely, when CU was at medium and high concentration (25-200 μmol/L), all the combination regimens exhibited high inhibiting activity on cell apoptosis. When the concentration of CU was less than 25 μmol/L, 1:2 group exhibited the strongest cell inhibition rate in SMMC-7721 cells.

In a word, the study was focus on new combination strategy to enhance anti-cancer effect of 5-FU. From the above results, the anti-cancer effect of 5-FU in SMMC-7721 cells could be enhanced by CU in a range of proportions. More concretely, 2:1 group showed the best synergy effect. So, the combination of CU and 5-FU with molar ratio of 2:1 and SMMC-7721 cells would be selected to further research the cell mechanism* in vitro* and pharmacodynamics* in vivo*.

### The mechanism of synergistic effect of joint group

**Figure 4:**
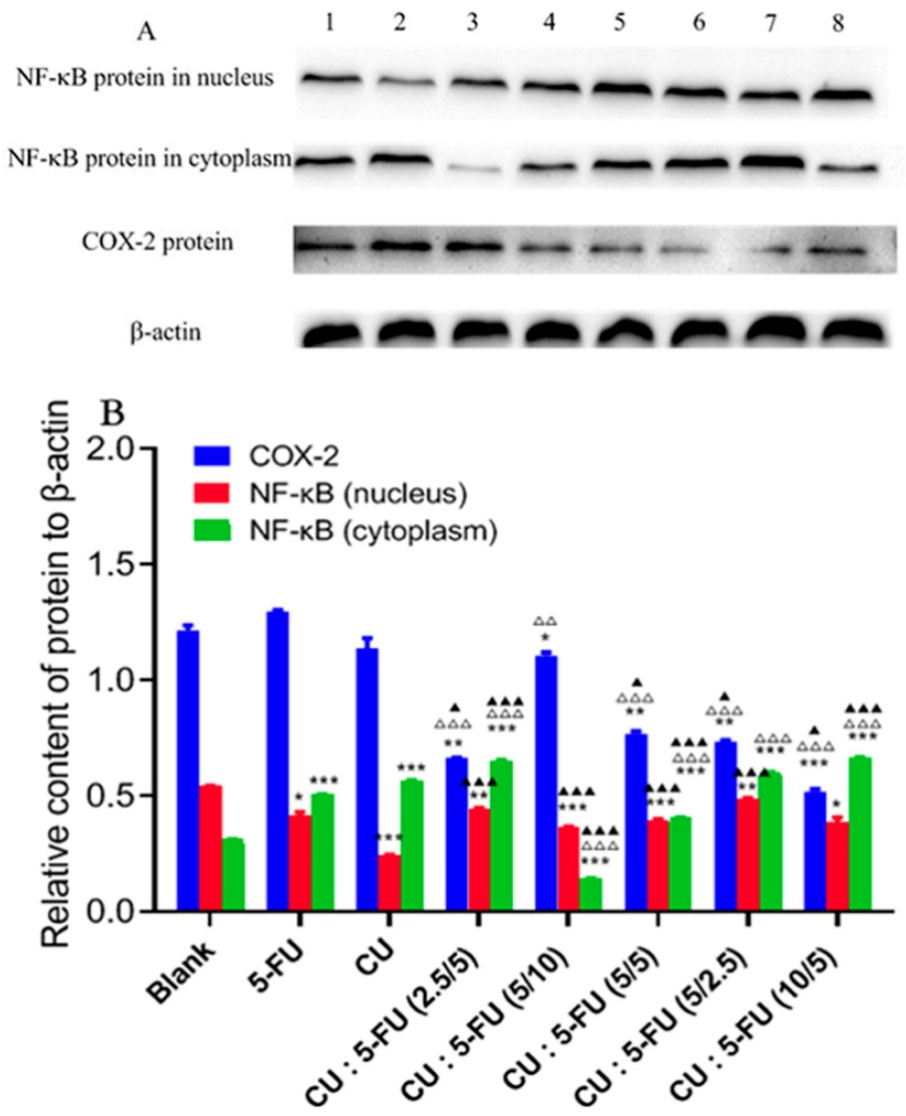
Effects of CU and 5-FU alone or in combination on the protein expressions of NF-κB in nucleus, NF-κB in cytoplasm and COX-2 on SMMC-7721 cells by Western blotting analysis. SMMC-7721 cells were treated with CU and 5-FU alone or in combination for 48 h. A: Band 1 was 15 μmol/L of 5-FU; Band 2 was 25 μmol/L of CU; Band 3 was 5 μmol/L of CU + 10 μmol/L of 5-FU; Band 4 was 5 μmol/L of CU + 5 μmol/L of 5-FU; Band 5 was 5 μmol/L of CU + 2.5 μmol/L of 5-FU; Band 6 was 10 μmol/L of CU + 5 μmol/L of 5-FU; Band 7 was 2.5 μmol/L of CU + 5 μmol/L of 5-FU; Band 8 was blank group. B: NF-κB and COX-2 protein expression in low- and high-concentration groups. The results are representative of at least three independent experiments run in triplicate and expressed as the means ± SEM. *P > 0.05 vs. the 5-FU group; P < 0.05 vs. groups among groups. *, p < 0.05, **, p < 0.01, and ***, p < 0.001, compared to the blank group; △, p < 0.05, △△, p < 0.01, and △△△, p < 0.001, compared to the 5-FU alone group; ▲, p < 0.05, ▲▲, p < 0.01, and ▲▲▲, p < 0.001, compared to the CU alone group.

The mechanism of SMMC-7721 cells inhibited by CU and 5-FU alone or combination was unclear. Therefore, western blotting analysis was used to detect the expression and variation trend of COX-2, the nucleus and cytoplasm NF-κB protein extracted from the total cell protein of SMMC-7721 cells. The results were shown in Figure 4. The intensities of protein bands of β-actin, COX-2, NF-κB in nucleus and cytoplasm are shown in Figure 4A. Compared with the blank group (Figure 4B), the expression of nucleus NF-κB protein was decreased while the cytoplasm NF-κB protein expression was increased after treated with the combination of CU and 5-FU for 48 h. The expression of nucleus NF-κB in blank group was higher than that in the single or combination groups. And the cytoplasm NF-κB expression in each combination group was higher than that in the 5-FU group except 1:2 group in high concentration and 1:1 group. Furthermore, the cytoplasm NF-κB expression of 2:1 group (mol/mol) was the highest. And the nuclear NF-κB expression of 2:1 group (mol/mol) was the lowest in all combination groups. On the one hand, as seen in Figure 4B, with the increase concentration of 5-FU (CU maintained at 5 μmol/L), the molar ratio of CU and 5-FU varied from 2:1 to 1:2, the expression of NF-κB protein in nuclear and cytoplasm increased. On the other hand, with the increase concentration of CU (5-FU maintained at 5 μmol/L), the molar ratio of CU and 5-FU varied from 1:2 to 2:1, the expression of nuclear NF-κB protein increased first and then decreased. However, the expression of cytoplasm NF-κB protein showed reverse rule. Thus, the combination of CU and 5-FU (2:1, mol/mol) showed great efficacy of inhibiting the transfer of NF-κB from cytoplasm to nucleus, especially CU: 5-FU (10/5) group.

As for COX-2 protein, it was shown that compared with control group, the COX-2 protein expression was down-regulated in all drug groups except 5-FU alone group. Simultaneously, the effect of down-regulating COX-2 protein in all combined groups was better than that in single groups. Among them, CU/5-FU (2:1) group in high concentration showed the best effect. Namely, at high concentration group, the expression of COX-2 in CU/5-FU (2:1) group was 2.3 folds lower than that in blank group, 2.2-2.5 folds lower than that in single group and 1.4-2.1 folds lower than that in other combined groups. At low concentration group, the expression of COX-2 in CU/5-FU (2:1) group was about 1.5 times lower than other groups. So, no matter in high or low concentration, 2:1 (mol/mol) group showed excellent advantages in inhibiting COX-2 expression.

Therefore, the mechanism of the inhibition efficacy of CU and 5-FU combination (2:1, mol/mol) on the proliferation of HCC cells may be related to inhibiting the transfer of NF-κB from cytoplasm to nucleus and down-regulation of COX-2 protein. However, its specific signal transduction pathway needed further study.

### *In vivo* antitumor study

**Figure 5:**
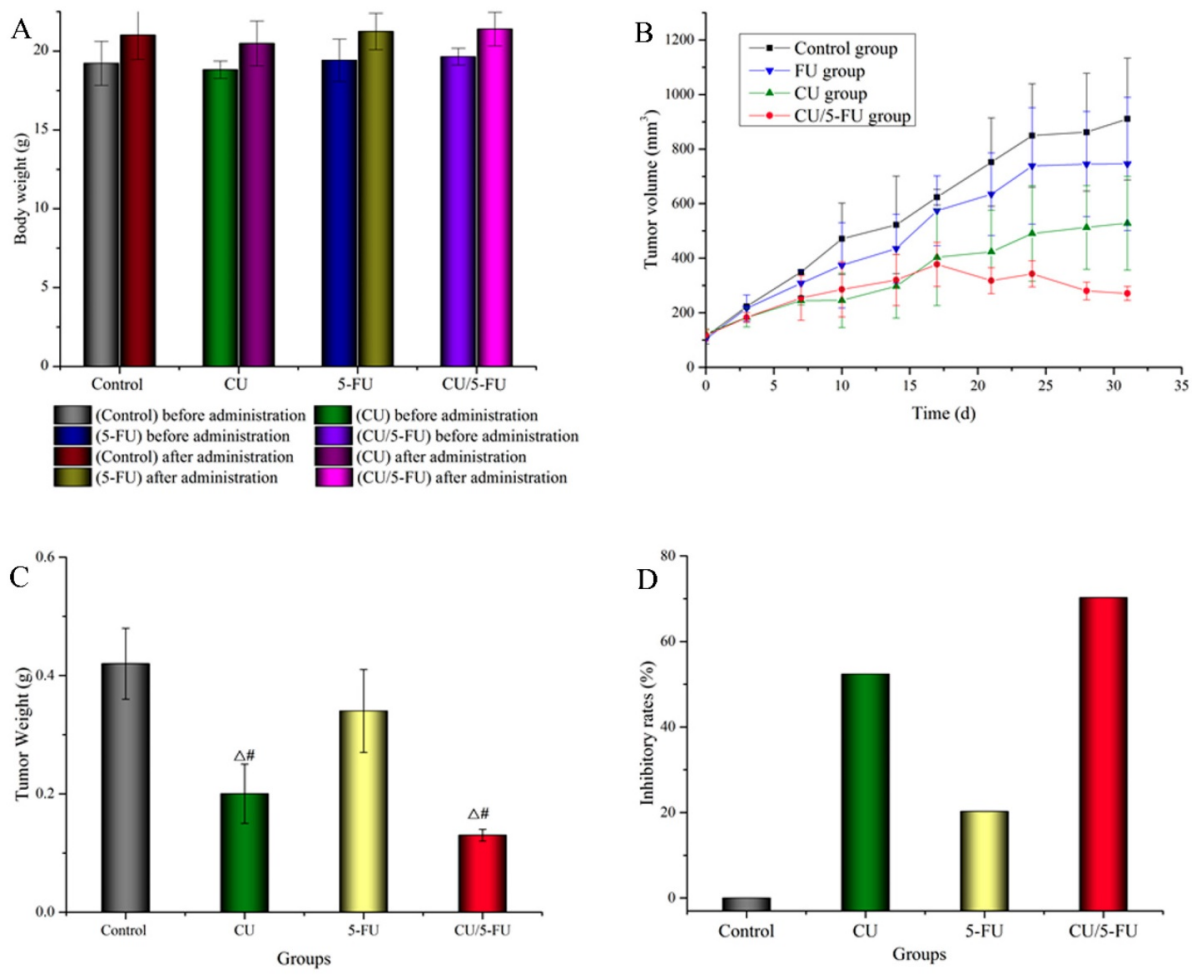
Antitumor efficacy and toxicity of CU and 5-FU alone or in combination in nude mice bearing SMMC-7721 tumor xenografts *in vivo*. A: The changes of body weight (g) before and after treated with CU and 5-FU alone or in combination in nude mice; B: The changes of tumor volume with different groups in nude mice; C: Tumor weight (g) of 7721-bearing nude mice treated with CU and 5-FU alone or in combination on day 31st when the mice were humanely sacrificed (mean ± SD). Note: T-test, ^△^*P* < 0.05 vs. the control negative group; ^#^*P* < 0.05 vs. the 5-FU group; D: Tumor inhibitory rates (%) of 7721-bearing nude mice treated with 5-FU and CU alone or in combination on day 31st.

During the experiment, the tumor-bearing mice in each group were in good mental state, and their diet and activities were normal. The changes of the body weight of mice, the volume of subcutaneous tumors, the tumors weight and the rate of inhibition of tumors were described in Figure 5A-5D, respectively. Figure 5A showed that the weight of nude mice in each group increased slightly before and after administration, and there was no significant difference between the administration group and the negative control group. Figures 5B and 5C showed that the growth of tumor in the negative control group was the most obvious. Compared with the negative control group, the growth of tumors in the other three groups tended to slow down, and the growth of tumors in CU/5-FU group was the slowest, which showed that the inhibitory effect of combined group on tumor growth was more obvious than that of single group. Figure 5D showed that compared with negative control group, all the other three groups had the effect of inhibiting the growth of tumors. The order of inhibition intensity was CU/5-FU> CU> 5-FU group. The results of the study *in vivo* indicated that the combination of CU and 5-FU (2:1, mol/mol) had synergistic effect on inhibiting the tumor growth of SMMC-7721-bearing mice, which verified the conclusion of cytotoxicity study.

## Discussion

Hepatocellular carcinoma (HCC) is the most common primary liver cancer with poor prognosis. However, the treatment options for advanced HCC are very limited. 5-FU-based chemotherapy was widely used for the treatment of HCC. However, the drug resistance and high toxicity limited the efficacy of 5-FU 33, 34. Therefore, it is necessary to find more effective chemotherapeutic drugs and/or combination therapy for HCC. At present, CU was widely researched due to its characteristics including inducing cell apoptosis, inhibiting tumor invasion and metastasis, reversing tumor resistance, etc 35. Yu et al. found that the growth of SMMC-7721 cells could be resisted by CU, which was due to inhibiting the Bcl-2 and activate Bax protein and promote caspase 3 pathway 36. And some researches also demonstrated that the anticancer effect of 5-FU against cancers could be enhanced by CU via down-regulation of COX-2 and NF-κB pathways 37, 38. Therefore, it may be an ideal strategy to use the combination of CU and 5-FU to treat HCC.

In this study, we evaluated the cytotoxic effect of 5-FU and CU alone or in combination with a various concentrations of 5-FU (6.25-200.00 µmol/ L) and CU (6.25-200.00 µmol/ L) against SMMC-7721 and Bel-7402 cells for 48 h drug exposure by MTT assay. The results demonstrated that the combination of 2:1 group showed stronger inhibitory effect on SMMC-7721 cells than that of 5-FU and CU alone with 10.1- and 20.6-fold increased cytotoxicity for 48 h treatment, respectively (Figure 1 & Table 1). And 2:1 group also showed the strongest synergistic effect in SMMC-7721 cells. Furthermore, the data were consistent with previous research that combination of SLN-curcumin and LDH-5-FU exhibited synergistic cytotoxic effects against SMMC-7721 cells 39. So these results indicated that combination administration of CU and 5-FU in certain proportional region may be a potent therapeutic regimen to treat HCC.

Study had shown that inhibiting the expression of NF-κB and COX-2 protein could increase the sensitivity of HCC and gastric cancer cells to chemotherapeutic drugs 40. In this research, the molar ratio (2:1) groups showed strongest inhibition of COX-2 in SMMC-7721 cells with statistical significance (P<0.05) (Figure 4B). Namely, 2:1 group inhibited the expressions of COX-2 compared to 5-FU alone and blank group with 50-60% inhibition whether in high or low concentration group, which was consistent with previous cytotoxicity studies. In addition, inhibiting NF-κB transferring from cytoplasm to nucleus can also improve the apoptosis of HCC cells. 2:1 group showed the great effect in decreasing the expression of NF-κB in the nucleus and increasing the expression of NF-κB in cytoplasma with concentration depending on SMMC-7721 cells (Figure 4B). Therefore, the greater inhibition of the combination of 2:1 group in SMMC-7721 cells compared to single drug alone may contribute, at least in part, to the inhibition of the expressions of COX-2 and NF-κB proteins.

To assess the synergistic effect of combination treatment against HCC *in vivo*, we examined whether the group of CU and 5-FU (2:1, mol/mol) inhibited the growth of the tumor of SMMC-7721-bearing nude mice. Before the experiment, the solvent used in the research was selected. Considering that CU was insoluble in water, proper solubilizer should be added to the solvent. Tween 80 and polyoxyethylene castor oil (EL) were the common solvents in injection. However, Tween 80 for injection had a high risk of hemolysis and anaphylaxis. The dosage of Tween 80 for injection was 0.5-1.0% but with low safety 41, 42. The most common and serious adverse reaction was complement activation-related pseudoallergy (CARPA), an acute hypersensitivity reaction, but CARPA could be slowly reduced or even disappeared over time 43, 44 Taxol, a paclitaxel injection, has been put into use in clinic. Its solvent was EL and absolute ethanol (1:1, v/v) 45. 5-FU in clinical application was dissolved in the sterile solution prepared by sodium chloride injection which adjusted pH with sodium hydroxide. So we investigated the dissolution of CU and 5-FU in the solution which adjusted pH with sodium hydroxide or sodium bicarbonate. The results showed that adjusting pH to weak alkalinity had no significant effect on the solubility of CU and 5-FU, and the solubility of drugs could not meet the dosage required in this study. After comprehensive consideration, EL-35: black absolute ethanol: sodium chloride injection (1:1:2, v/v/v) was used as the solvent to dissolve CU and 5-FU to reach the desired concentration. And the pre-experiments showed that nude mice had good tolerance to this injection. After the experiment, the weight of mice did not decrease (Figure 5A). The volumes (Figure 5B) of SMMC-7721 tumors in mice treated with vehicle, CU or 5-FU alone were gradually increased with time increase. However, the tumor volume in mice treated with CU plus 5-FU was decreased. In addition, the results of tumor weight (Figure 5C) and tumor inhibition rate (Figure 5D) also showed that the effect of combination was stronger than that of single and control group. The results of pharmacodynamics *in vivo* were in agreement with that of cytotoxicity *in vitro*, which indicated that the combination of CU and 5-FU (2:1, mol/mol) showed synergistic effect in inhibiting HCC.

## Conclusion

In conclusion, the combination of CU and 5-FU showed obvious synergistic effect in a certain proportion including 1:1, 1:2, 2:1 and 4:1 (ratios of CU and 5-FU) on SMMC-7721 cells. Among them, the optimal ratio of combined anti-hepatocellular carcinoma was 2:1 (CI < 0.3). And the 2:1 group also showed excellent effect on inhibiting the growth of tumors in the nude mice with SMMC-7721 subcutaneous tumors, which was consistent with the results of *in vitro* experiments. The mechanism of that may be related to the inhibition of the expression of COX-2 and the reducing the transfer of NF-κB from cytoplasm to nucleus.

## Figures and Tables

**Table 1:** The IC_50_ values of CU and 5-FU alone and in combination in L02, SMMC-7721, Bel-7402, HepG-2 and MHCC97H cells for 48 h.

CU/5-FU (mol/mol)	Drugs	IC_50_ (μmol/L)				
		L02	SMMC-7721	Bel-7402	HepG-2	MHCC97H
1:01	CU	34.08 ± 2.36	4.95 ± 1.20	19.30 ± 2.29	25.00±1.21	35.35 ± 2.84
	5-FU	34.08 ± 2.36	4.95 ± 1.20	19.30 ± 2.29	25.00±1.21	35.35 ± 2.84
1:02	CU	15.06 ± 0.66	2.49 ± 0.58	16.15 ± 2.01	27.34±1.72	16.04 ± 1.12
	5-FU	30.12 ± 1.32	4.93 ± 1.17	32.3 ± 4.02	54.68±3.45	32.07 ± 2.25
1:04	CU	32.1 ± 1.46	6.15 ± 1.10	7.63 ± 1.31	31.07±0.84	22.09 ± 1.19
	5-FU	128.4 ± 5.83	24.60 ± 4.40	30.52 ± 5.24	124.27±3.37	88.36 ± 4.77
2:01	CU	26.45 ± 1.92	4.32 ± 2.02	45.60 ± 2.56	10.36±1.77	14.48 ± 1.26
	5-FU	13.23 ± 0.96	2.16 ± 1.05	22.80 ± 1.78	5.17±0.88	7.24 ± 0.63
4:01	CU	9.33 ± 0.81	17.72 ± 4.68	17.72 ± 3.88	23.59±4.51	25.78 ± 2.58
	5-FU	2.33 ± 0.20	4.28 ± 1.17	4.43 ± 0.97	5.90±1.13	6.44 ± 0.64
-	CU alone	22.46 ± 2.69	89.06 ± 11.85	94.74 ± 4.03	43.46±5.34	65.27 ± 2.22
	5-FU alone	32.98 ± 1.23	21.90 ± 1.54	38.48 ± 2.27	40.18±6.24	43.86 ± 1.99

**Table 2:** The CI values of CU and 5-FU alone and in combination in cells for 48 h.

CU/5-FU (mol/mol)	Cell lines				
	L02	SMMC-7721	Bel-7402	HepG-2	MHCC97H
1:1	2.62	0.24004	0.60773	2.14	1.96
1:2	0.78	0.15843	0.81156	0.94	1.14
1:4	1.17	1.1316	0.69721	1.48	3.04
2:1	3.01	0.14698	0.43735	0.67	0.43
4:1	1.6	0.36951	0.285	0.62	0.51
